# An anxiety management intervention for people with substance use disorders (ITASUD): An intervention mapping approach based on Peplau's theory

**DOI:** 10.3389/fpubh.2023.1124295

**Published:** 2023-02-21

**Authors:** Caroline Figueira Pereira, Divane de Vargas, Linda S. Beeber

**Affiliations:** ^1^Mental Health and Psychiatric Nursing, School of Nursing, University of São Paulo, São Paulo, Brazil; ^2^School of Nursing, University of North Carolina at Chapel Hill, Chapel Hill, NC, United States

**Keywords:** intervention mapping, anxiety, Peplau's theory, substance use disorders, evidence-based interventions, anxiety management

## Abstract

**Background:**

The comorbidity of anxiety and drug use disorders complicates treatment prognosis, and one of the greatest challenges is to address the environmental and behavioral factors involved. The aim of this study was to describe the uses of intervention mapping in the design of a theory and evidence-based complex intervention to develop skills around the management of anxiety for cocaine users in outpatient addiction treatment.

**Methods:**

The six steps of the intervention mapping approach, which are needs assessment, creation of matrices of performance objectives, selection of methods and practical strategies, program development, adoption and implementation, and evaluation were applied to develop the Interpersonal Theory of nursing to Anxiety management in people with Substance Use Disorders (ITASUD) intervention. The theory used for the conceptual model was interpersonal relations theory. All theory-based methods and practical applications were developed at the individual level, acting in behavioral, interpersonal, organizational and community environments.

**Results:**

The intervention mapping provided a broad overview of the problem and outcome expectations. The ITASUD intervention consists of five consecutive sessions of 110-min targeting individual determinants of anxiety (knowledge, triggers, relief behaviors, self-efficacy and relations), delivered by a trained nurse using Peplau's concepts of interpersonal relations. Intervention Mapping is a multi-step process that incorporates theory, evidence, and stakeholder perspectives to ensure that implementation strategies effectively address key determinants of change.

**Conclusions:**

The intervention mapping approach increases the effectiveness of the intervention since the matrices provide a broad view of all factors that affect the problem and facilitate replication through transparency of the determinants, methods, and applications used. ITASUD addresses all factors that play an important role in substance use disorders based on a theoretical basis, which provides the translation of evidence from research into effective practice, policy, and public health improvements.

## Background

The comorbidity of anxiety with drug use disorders has been investigated by several researchers (1), and a strong association has been shown. This association complicates treatment prognosis and increases services utilization and health care costs, generating a global issue. The main challenges are to address both anxiety and substance use disorders by identifying the relationship between them and the environmental and behavioral factors involved.

Anxiety is one of the most prevalent health problems worldwide, generating health care costs, a high burden of disease, and implications associated with untreated illness that affect health, economic, and social sectors (2). According to the World Health Organization (3), the consequences of anxiety disorders occupy the 6th position in the ranks of contributors to global disability. Clinical reviews have shown that the presence of an anxiety disorder is a risk factor for the development of mood disorders and substance abuse (1, 2) and is highly comorbid with other mental disorders (4, 5).

Research has shown a 14.9% increase since 2005 in the number of people living with anxiety (6), and 21% of that estimated number is in the region of the Americas (3). In Brazil, the prevalence of anxiety disorders is 9.3% (3) of the population, and another factor that has been shown to be related to this prevalence is the increase in cocaine users in the country. Currently, cocaine is the most commonly used stimulant in Brazil. One explanation is that Brazil is the largest cocaine market in South America, because of its geographic position (neighboring the world's largest cocaine producers—Peru, Colombia and Bolivia), and leader in the cocaine trafficked from South America to Africa, Europe and Africa (6). A previous study (7) showed that the prevalence of cocaine users in the country is ~3.2 million people. This high prevalence occasioned an increase in cocaine users arriving in emergency care, resulting in Brazilian cocaine users arriving in emergency care at three times higher rates than elsewhere in the world (8, 9). This high demand in emergency care demonstrates that it is necessary to optimize treatment in specialized facilities for people with substance use disorders to avoid this high demand in emergency care.

The major challenge with this population is keeping them in specialized treatment, as high levels of anxiety are the main cause for relapse and withdrawal from treatment. One of the keys for keeping this population in treatment is to treat the anxiety experienced by cocaine users. This article presents the development of an implementation intervention called the Interpersonal Theory of nursing to Anxiety management in people with Substance Use Disorders (ITASUD) that can be incorporated into daily health care programs in early intervention focusing on the management of anxiety in cocaine users with a focus on the steps of the intervention mapping (IM) approach. These steps of IM are guided by theory, evidence, and input from relevant stakeholders perspectives to improve the effectiveness and ensure that implementation strategies effectively address key determinants of change (10, 11). Since, according to studies (12, 13), the implementation strategies often were poorly conceived, with incongruence between strategies and determinants, and the effect is variable and tends to be small to moderate; it remains unclear how determinants should be identified, decisions should be made on which determinants are most important to address, and strategies should be selected to address the important determinants (13). This signals a need for more rigorous processes and methods to guide these key steps of implementation strategy selection and tailoring (10–14).

## Peplau's theory

The Peplau's theory provides the concepts that guide in the establishment of a strategic communication with clients by using an observational, experiential, and reflexive approach in structured and unstructured interactions. A central driver of the interpersonal relationship process developed by Peplau is anxiety, which, if strategically approached, can be a key to clients' health problems (15). Anxiety is defined by Peplau as a tension that generates energy transformation, this energy transformation generates physiological and behavioral answers. The main way to work with the tension of anxiety is to learn how to be aware of it and enact strategies that keep the level of anxiety in the mild to moderate range, which allows it to power productive growth. However, the tension of anxiety is often neglected in the interpersonal relationship due to the lack of theoretical knowledge and research addressing anxiety, mainly in the treatment of individuals with substance use disorders. The use of psychoactive substances is a kind of relief behavior used to decrease anxiety, and it can be transformed into a pattern of behavior that changes the self-system. Therefore, to treat people with substance disorders, it is necessary to treat the anxiety felt by them, because it is this anxiety that leads to the initiation of psychoactive substances and that plays an important role in relapse, and to keep patients in treatment.

## Method

The methodology used to develop the intervention was the systematic IM (16) development process. IM is a framework consistent with the Medical Research Council (17) guidance on developing complex interventions and has been used to develop intervention programs for many health behaviors (18–23) because it employs an ecological approach that considers environmental influences on behavior and develops methods and strategies to address them (18).

IM is very useful because it specifies processes for integrating theoretical constructs and evidence-based literature for the purposeful development of an intervention through a description of a logical planning process. IM is a six-step process that is structured and sequenced as follows: (1) needs assessment (logic model of the problem); (2) creation of matrices of performance objectives (logic model of change); (3) selection of theory-based methods and practical strategies (program design); (4) program development; (5) adoption and implementation; and (6) evaluation.

### Step 1: Needs assessment

The aim of step 1 is to develop the logic model of the problem, which allows for the programming of goals for the intervention related to health and quality of life. The logic model in the present study was based on the combination of a comprehensive understanding of the problem through Peplau's theory, empirical data about the factors that contribute to the problem, and experiential information about the problem. In addition, this step focuses on the description of the intervention context (population, setting and community).

### Step 2: Creation of matrices of performance objectives

Step 2 of IM involves the following: (1) developing a statement of expected outcomes related to behavior and the environment, and developing performance objectives related to those behavioral and environmental outcomes; (2) identifying selected determinants related to the behavioral and environmental outcomes; and (3) constructing matrices of change objectives and creating the logic model of change.

The main aim of this step is the development of the logic model of change, which represents pathways of the intervention that act from behavioral and environmental perspectives through the connections between determinants and change objectives, performance objectives, desired outcomes, and improved quality of life in relation to the health problem of anxiety.

### Step 3: Selection of theory-based methods and practical strategies

Step 3 is to generate program themes, components, scope and sequence. To accomplish this aim, we chose Peplau's theory as a conceptual model and method and selected evidence-based methods to reach change objectives. We also used published guidance on the intervention mapping approach (16) to choose some methods based on the definition and parameter of each method.

### Step 4: Program development

Guided by the matrices, the team started to refine program structure and organization, prepare plans for program materials, and develop specific messages, materials and protocols. The change objectives were converted into practical applications using a range of evidence-based research. At the end of this step, the definitive intervention content and materials were created based on relevant additions made through the team discussion.

### Step 5: Adoption and implementation

The aim of the program implementation plan was to determine the balance between what was planned and what could be implemented in the real world through the identification of potential users (adopters, implementers and maintainers) and the context in which the users are inserted, resulting in a better implementation design. Additionally, an intervention manual was adapted to increase the chances of adoption, implementation and sustainability.

### Step 6: Evaluation

After step 5, adoption and implementation, this program was evaluated through operational definitions of feasibility, such as acceptability, demand, practicality, and adaptation (24).

The qualitative data were analyzed using thematic analysis described by Braun and Clarke (25) for identify, analyze, and reporting patterns within data. The phases of thematic analysis were: (1) familiarizing with the data—transcription of the data, reading and re-reading the data, noting down initial ideas; (2) generate initial codes—coding interesting features of the data across the entire data set, collating data relevant to each code; (3) searching for themes—collating codes into potential themes, gathering all data relevant to each potential theme; (4) reviewing themes—checking if the themes work in relation to the coded extracts and the entire data set, generating a thematic “map” of the analysis; (5) defining and naming themes—generating clear definitions and names for each theme; (6) producing the report—final analysis of selected extracts.

Although the feasibility study is a component of step 6, the evaluation of this intervention program is not within the scope of the current paper; the feasibility study will be only briefly discussed in the Results and Discussion sections.

## Theoretical approach

According to IM, it is important to use a theory and evidence to specify determinants and behavioral and environmental factors that are related to the health problem that the intervention intends to address. The present intervention used Peplau's Interpersonal Theory of Nursing (ITN) (15) as the conceptual model, and we selected some methods associated with other theories to achieve certain intervention outcomes based on empirical findings. The ITN is a middle-range theory used for nursing in psychotherapeutic intervention, and we used some concepts from that theory as determinants of anxiety, such as knowledge, triggers, relief behaviors, self-efficacy and relations.

## Results

### Intervention development

#### Step 1: Needs assessment

The priority population is adult male cocaine users (age > 18 years) with anxiety who are residents in Brazil and undergoing treatment in specialized outpatient health facilities. We are focusing on male cocaine users due to the higher prevalence of crack use by adults, especially among young males (26–30), and because we understand that there are differences between the sexes in brain chemistry, physiology and the way that they tend to deal with stress and anxiety (1). In Brazil, cocaine users have been identified as a major health and social problem owing to the increase in users presenting at health facilities and the increase in illegal activities affecting urban security (30).

Based on the theoretical explanation provided by Peplau's theory (15), cocaine use is a kind of relief behavior used to decrease anxiety, and it can be transformed into a pattern of behavior that changes the self-system. Therefore, to treat cocaine users, it is necessary to treat the anxiety felt by cocaine users, because it is this anxiety that led to the initiation of cocaine use and that plays an important role in relapse, and to keep patients in treatment.

**Figure 1:**
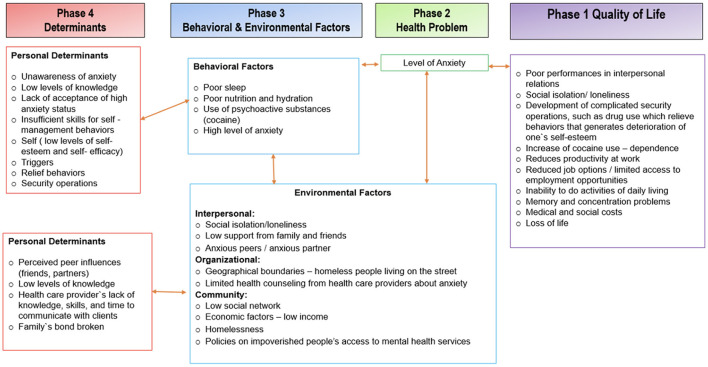
Logic model of problem.

We developed a logic model of the problem to connect all behavioral and environmental factors that play an important role in anxiety, including its determinants and its consequences on quality of life (Figure 1).

#### Step 2: Creation of matrices of performance objectives

**Figure 2:**
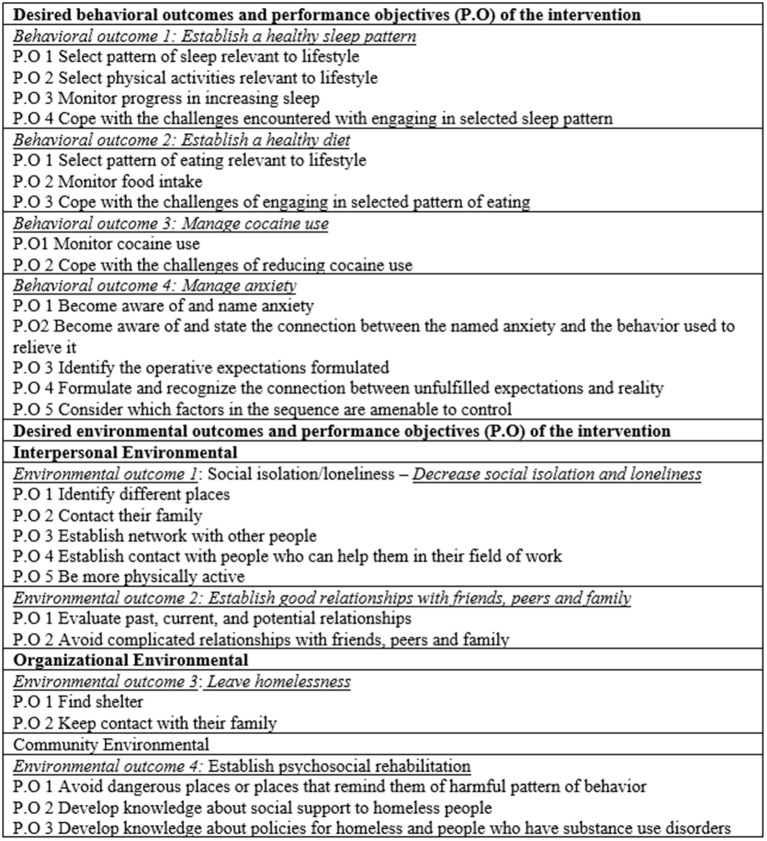
Expected outcomes for behavioral and environmental outcomes.

To achieve the first step of step 2, we worked from the needs assessment and an integrated theoretical framework to specify behaviors and environmental conditions that the program would promote using the logic model of the problem (Figure 1) as a guide to develop desired behavioral and environmental outcomes and then create performance objectives for each behavioral and environmental outcome (Figure 2).

The determinants (knowledge, triggers, relief behaviors, self-efficacy, and relations) were developed by adapting Peplau's concepts from a behavioral and environmental perspective (31). The first step of this adaptation was to choose some of Peplau's concepts that are related to anxiety and substance abusers to develop relational propositions among them.

**Figure 3:**
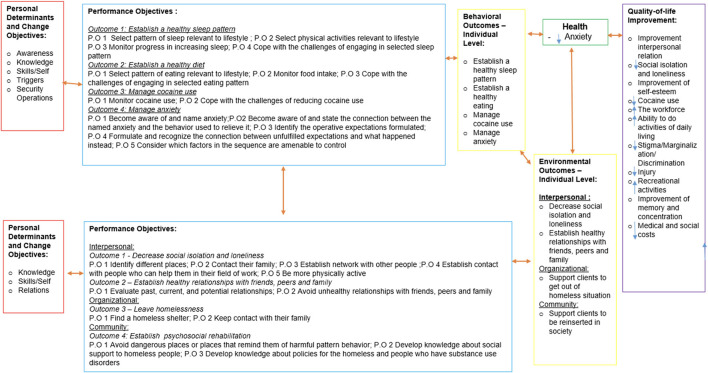
Logic model of change.

Finally, we integrated the behavioral and environmental outcomes with performance objectives and determinants. The first thing that we developed was the logic model of change (Figure 3), and through this model, we constructed the matrices of change objectives based on each behavioral and environmental outcome (Table 1). For example, the behavioral outcome “establish a healthy sleep pattern” has four determinants (knowledge, triggers, relief behaviors, and self-efficacy), and for each determinant, we wrote a performance outcome (specific outcome) that would be expected to occur as a result of the intervention.

**Table 1:** Matrices construction of change objectives.

**Performance objectives**	**Knowledge**	**Triggers**	**Relief behaviors**	**Self-efficacy/self- esteem and skills**
**Behavior: Establish a healthy sleep pattern**				
P.O 1 Select pattern of sleep relevant to lifestyle	K.1 Develop knowledge about the most common patterns of sleep	T.1 Analyze the triggers that interfere with sleep	RB.1 Create new relief behavior to achieve sleep	SES.1 Build self-confidence in ability to adequate pattern of sleep in their lifestyle
P.O 2 Select physical activities relevant to lifestyle	K.2 Develop knowledge of the benefits of physical activities to improve sleep	T. 2 Identify triggers that interfere with physical activity	RB.2 Identify physical activity as a relief behavior	SES.2 Build self-confidence in ability to do physical activity
P.O 3 Monitor progress in increasing sleep	K.3 Develop knowledge about their habitual pattern of sleep	T.3 Evaluate triggers that interrupt sleep	RB.3 Distinguish the relief behaviors that promote healthy sleeping habits from those that impede them	SES.3 Build self-confidence in ability to change pattern of sleep
P.O 4 Cope with the challenges of engaging in selected pattern of sleep	K.4 Develop knowledge of the challenges to achieve a good pattern of sleep and safer places to sleep	T.4 Avoid triggers that interrupt sleep	RB.4 Modify the relief behaviors that are used to achieve sleep	SES.4 Build self-confidence in ability to face the challenges of adopting healthy sleeping pattern
**Behavior: Establish a healthy diet**				
P.O 1 Select pattern of eating relevant to lifestyle	K.1 Develop knowledge about the possible pattern of eating in their lifestyle	T.1 Analyze triggers that interfere in the healthy diet	RB.1 Develop new relief behavior to achieve a good diet	SES.1 Build self-confidence to adjust eating patterns to their lifestyle
P.O 2 Monitor food intake	K.2 Develop knowledge about the healthier pattern of eating	T.2 Evaluate the triggers that affect the diet	RB.2 Distinguish the relief behaviors that promote from those that impede a healthy diet	SES.2 Build self- confidence to monitor one's own food intake
P.O 3 Cope with the challenges of engaging in selected healthy eating pattern	K.3 Develop knowledge about the places to eat healthily	T.3 Avoid the triggers that interfere with healthy eating	RB.3 Modify the relief behaviors to achieve a healthy diet	SES.3 Build self-confidence to face the challenges encountered toward pattern of eating
**Behavior: Manage cocaine use**				
P.O 1 Monitor cocaine use	K.1 Develop knowledge about the habitual consumption of cocaine and its characteristics	T.1 Analyze the triggers that interfere directly or indirectly in cocaine use	RB.1 Develop new relief behaviors	SES.1 Build self-confidence to monitor one's own cocaine use
P.O 2 Cope with the challenges of manage cocaine use	K.2 Develop knowledge about the challenges to manage consumption of cocaine	T.2 Avoid the triggers that influence in the cocaine use	RB.2 Modify the relief behaviors	SES.2 Build self-confidence to generate coping strategies to deal with the challenges
**Behavior: Manage anxiety**				
P.O 1 Become aware of and name anxiety	K.1 Develop knowledge about what anxiety means	T.1 Identify the triggers of anxiety	RB.1 Identify the relief behaviors used during anxiety	SES.1 Build self-confidence to recognize anxiety
P.O 2 Become aware of and state the connection between the anxiety and behaviors to relieve it	K.2 Develop knowledge about the connection between the anxiety and behaviors to relieve it	T.2 Define the connection between triggers and anxiety	RB.2 Analyze the relief behaviors	SES.2 Build self-efficacy to establish connection between anxiety and relief behaviors
P.O 3 Identify the expectations created	K.3 Develop knowledge about the influence of expectations	T.3 Analyze the triggers involved in the operative expectations	RB.3	SES.3 Build self-confidence to recognize expectations
P.O 4 Recognize the connection between unfulfilled expectations and what happened instead	K.4 Develop knowledge about the connection between unfulfilled expectations and what happened instead	T.4 Appraise the connection among triggers, expectations and what happened instead	RB.4 Identify whether the relief behaviors are connected with the expectations	SES.4 Build self-efficacy to identify the connection among expectations, what happened instead, anxiety and relief behaviors
P.O 5 Consider which factors in the sequence are amenable to control	K.5 Develop knowledge about the connection between the controllable factors and anxiety	T.5 Categorize the factors in possible triggers for anxiety	RB.5 Modify the relief behaviors that are changeable	SES. 5 Build self-confidence to change expectations and factors to break the cycle
**Interpersonal environmental: Decrease social isolation and loneliness**			
P.O 1 Identify different places to reduce loneliness and social isolation	K.1 Develop knowledge about accessible locations for social interaction near the place where the client lives	R.1 Prepare to establish interpersonal bonds in new places	SES.1 Build self-efficacy to recognize barriers‘ to knowing different places
P.O 2 Contact family	K.2 L Develop knowledge about family's story	R.2 Prepare to reestablish relational bonds	SES.2 Build self-confidence to establish contact with their family
P.O 3 Establish network with other people	K.3 Develop knowledge about the importance of healthy network	R.3 Establish interpersonal bonds in new places	SES.3 Build self-confidence to establish healthy networks
P.O 4 Establish contact with people who can help them with job opportunities	K.4 Develop knowledge about the importance of a network that can help them with job opportunities	R.4 Find healthy networks with other people	SES.4 Build self-confidence to contact people who can help them with job opportunities
P.O 5 Be more physically active	K.5 Develop knowledge about physical activity programs in different places near the place they live	R.5 Establish relational ties to support physical activity	SES.5 Build self- confidence to practice physical activity
**Interpersonal environment: Establish good relationships with friends, peers and family**			
P.O 1 Evaluate past, current, and potential relationships	K.1 Develop knowledge about the past, present and future relationships	R.1 Deep understanding about interpersonal relations that are significant (relational bonds and ties)	SES.1 Build self-confidence to differentiate good and complicated relationships
P.O 2 Avoid complicated relationships with friends, peers and family	K.2 Develop knowledge and strategies about how to avoid problematic relationships	R.2 Recognize complicated relational bonds and ties	SES.2 Build self-confidence in their ability to change/ finish complicated relationships
**Organizational environment: Leave homelessness**			
P.O 1 Find a shelter	K.1 Develop knowledge about the shelter available in their environmental scenario and the shelter's rules	R.1 Identify shelter as a positive relational tie	SES.1 Build self-confidence to find a better shelter for their reality
P.O 2 Keep contact with their family	K.2 Develop knowledge about the importance of family	R.2 Synthesize the dynamic of family‘s bond	SES.2 Build self-confidence to keep contact with their family and return to their family‘s house, if possible
**Community environment: Establish psychosocial rehabilitation**			
P.O 1 Avoid dangerous places or places that remind them of harmful pattern behavior	K.1 Develop knowledge about the advantages to avoid dangerous places or places that remind them of harmful pattern behavior	R.1 Deep understanding about the relations in dangerous places	SES.1 Build self-confidence to deal with environmental cues
P.O 2 Develop knowledge about social support to homeless people	K.2 Develop knowledge about the social support available in their city	R.2 Establish relational ties to achieve social support	SES.2 Build self-confidence to visit social support in their region
P.O 3 Develop knowledge about policies for the homeless and people who have substance use disorders	K.3 Develop knowledge about the specific policies for the homeless and people who have substance use disorders available in their city	R.3	SES.3 Build self-confidence to search some important policies

#### Step 3: Selection of theory-based methods and practical strategies

The first task was to organize all the change objectives created in step 2 (Table 1) together with the performance objectives, according to the determinants which they were associated. The second task was to match change objectives with specific determinants and methods selected. In the third task, the team started to propose applications, that is, strategies to operationalize the delivery of these methods, for each change objective (Supplementary File 1, Step 3).

#### Step 4: Program development—ITASUD

The selected theories that we used to develop the intervention were behavioral cognitive theory (BCT) (32), Peplau's theory (15), social cognitive theory (33), the trans-theoretical model (34), goal-setting theory (35), theories of information processing, the precaution adoption process model (36), self-affirmation theory (37), theories of automatic, impulsive and habitual behavior (38), attribution theory and relapse prevention theory (39), theories of goal-directed behavior (40), theories of social networks and social support (41), and theories of self-regulation (42). Consensus regarding the theories, methods, and applications was built for final agreement based on the adaptations needed for the intervention prototype structure to provide and equip cocaine users with the tools to manage anxiety in specialized outpatient health facilities.

The program ITASUD (Interpersonal Theory of nursing to Anxiety management in people with Substance Use Disorders) was designed for individual sessions (nurse-client) based on the phases of interpersonal relations proposed by Peplau (orientation, work and resolution) during five consecutive sessions delivered by a trained nurse using Peplau's concepts of interpersonal relationships. The first session would last 30 min, and the subsequent sessions would last 20 min. We chose this duration of time because in Brazil, according to public health minister (43), nurses need to attend three clients in a period of 60 min. However, the first session is scheduled for longer than the others because this session consists of two phases of interpersonal relationships (orientation and work).

The prototype of the intervention manual was guided by the matrices and the ideas about the methods and strategies that make possible the construction of specific messages and the overall content of each program component. During the intervention manual phase, we used the matrices developed during phase 3 and included the structure of each appointment; that is, we thought about the order of the target behavioral and environmental outcomes and the time and material used. All material used, such as notebooks, guidelines, and images, was designed to be appropriate for individuals with low literacy skills and was produced in English and Portuguese.

#### Step 5: Adoption and implementation of ITASUD

The outcomes of the program were divided into behavioral—to establish a healthy sleep pattern, to establish healthy eating, to manage anxiety, and to manage cocaine use—and environmental outcomes—to decrease social isolation, to have healthy relationships, to get out of homeless situations, and to be reinserted into society. However, these behavioral and environmental outcomes were developed at the individual level. During the outcome development, the reality of the population (homeless people and cocaine/crack users) and how this reality could affect the program were considered. The authors structured the intervention sessions as self-contained due to the potential high rate of dropout. We thought that in this specific population, the client could come to the first session and may not return for the other sessions, and therefore, the minimal dose will be one appointment and the maximum dose will be five appointments.

#### Step 6: ITASUD evaluation

To evaluate the intervention manual, we conducted a focus group with nurses who worked in a health facility to deeply understand their conceptions about the intervention plan to equip cocaine users to manage anxiety through specific questions that addressed each operational definition of feasibility through a guideline to conduct the focus group (Supplementary File 2).

The focus group was composed of seven nurses who worked in a specialized outpatient health facility in São Paulo. The nurses who participated were composed by four women (57.14%) and three man (42.85%); the majority were married (*n* = 5; 71.42%), white (*n* = 6; 85.71%), and to practice a religion (*n* = 5; 71.42%). We used thematic analysis to investigate the themes (25). Twelve themes related to feasibility adaptation were generated (*n* = 5; different world, environmental factor as the most important, relation between behavior and environmental factors, food, and specialized outpatient health facilities' focus on the anxiety), demand (*n* = 3; intoxication level, exclusion criteria, and clients who use crack/cocaine), acceptability (*n* = 1; answering the scale), and practicability (*n* = 3; worries about applying the intervention, placing the appointment, and adequate time in the work day).

The most frequent theme was related to adaptation of the intervention to the specificity of the particular population being studied (homeless, illiterate, vulnerable) and the factors that could influence the acceptance of the intervention to decrease the rate of treatment withdrawal. The nurses' opinions were mixed about the scale application in the first and last appointments, mainly because of the large number of scales; some nurses said that once the patient accepted participation in the intervention, they would answer the scales regardless of the number, but other nurses said that the intervention involves too many scales to ask a patient who presents with a high level of anxiety to answer. All nurses considered it important to change the order in which the environmental and behavioral factors that affect anxiety were addressed approached. They suggested approaching environmental situations first and then the behavioral factors, and they suggested taking out two of the identified behavioral factors, sleeping and eating patterns, because of the homeless situation of the participants in the intervention.

In relation to practicability, they raised issues such as the times for the appointment and the complexity of the intervention manual; in particular, all nurses agreed that 20 min to address all the issues related to anxiety in each appointment could be insufficient. The main theme related to demand was intoxication status because the health facility is an outpatient facility, and it is difficult for patients to remain abstinent, mainly at the onset of treatment. After the focus group, the team altered the intervention manual to improve the intervention feasibility for this particular health care facility scenario.

We described above some data from feasibility studies related to the development of the intervention, mainly the intervention manual, to achieve step six. The participants' recruitment process of the feasibility trial was by convenience sampling, totalizing 39 participants in the feasibility trial, what is considered enough for a feasibility study (44). The data was analyzed by the use of the linear mixed effects model to assess the changes in the level of anxiety after the intervention. The feasibility trial indicated that ITASUD appears to be feasible and support the design of a powered larger trial to evaluate the effectiveness of the ITASUD (45).

## Discussion

This study describes the development of an intervention to equip cocaine users with strategies to manage anxiety through the IM approach. There is limited literature about the phases of the development of complex interventions, mainly in this theme that includes comorbidities (anxiety and cocaine use). One of the explanations for this sparse literature is that the development of complex interventions is a considerable challenge for researchers, mainly because it is very difficult to identify all the factors that play an important role in the health problem of interest and to present all the steps of a new intervention in such a way that the readers can understand the entire process undertaken to develop the intervention protocol.

IM is based on the creation of matrices to show all the steps in the development of the intervention. The process of creating these matrices is very time consuming but helps the authors of all components during the brainstorming to see the relationships between the determinants, change objectives, performance objectives, and behavioral and environmental outcomes that affect the health problem being studied. In addition, this methodological process assists in the development of the theoretical framework of the intervention, which is necessary for the identification of mediators and moderators of the intervention that will play an important role in the success and failure of any particular intervention.

The needs assessment produced during this study was fundamental to identify the barriers to accessing cocaine users and services based on the logic related to this population of outpatients, such as high rates of dropping out, undocumented status, and environmental factors that directly affect the level of anxiety. IM is a powerful methodological tool that facilitates the comprehensive examination of these environmental factors using an ecological perspective instead of acting only on behavioral factors. We followed all the steps of IM using a mix of quantitative and qualitative methods to achieve a more comprehensive intervention manual that incorporates resources that affect the majority of the identified barriers to accomplish the desired outcomes of the intervention.

The overall structure of the program was adapted to the particular patient population based on the stakeholders' experiences. The structure of the intervention was adapted such that environmental factors are addressed in the 4th appointment rather than the 5th appointment, and this change was taken to enhance clients' acceptability: as demonstrated in the focus group, all nurses said that the environmental factors were the factors that the clients liked to talk about, which would affect each individual's level of anxiety. Additionally, we removed two behavioral factors, sleeping and eating, that we identified as important factors in increasing anxiety, but during the focus group, all nurses identified these factors as difficult topics to broach with the clients, as the majority of them were homeless. The intervention will be applied over five consecutive days, 20 min for each appointment, while taking into consideration the high dropout rates of these clients in the outpatient service.

## Conclusion

This study will provide valuable guidance for future researchers, health agencies, and health care professionals who are interested in reproducing this systematic approach to developing a complex intervention, once there is limited literature about the phases of the development of complex interventions. The program has already been implemented in the feasibility study, and demonstrate to be feasible. Additionally, ITASUD can be generalizable to other settings around the world into daily care programs.

## Data availability statement

The original contributions presented in the study are included in the article/Supplementary material, further inquiries can be directed to the corresponding author.

## Ethics statement

Ethical approval was obtained from the Institutional Review Board of the School of Nursing, University of São Paulo (CAEE number: 86848418.4.0000.5392) and the Municipal Health Secretary of São Paulo (CAEE number: 86848418.4.3001.0086). The patients/participants provided their written informed consent to participate in this study.

## Author contributions

All authors listed have made a substantial, direct, and intellectual contribution to the work and approved it for publication.
