# Supplementary material for: An anxiety management intervention for people with substance use disorders (ITASUD): An intervention mapping approach based on Peplau's theory
Source: Front Public Health. 2023 Feb 21;11:1124295. doi: 10.3389/fpubh.2023.1124295 (PMC9989484; doi:10.3389/fpubh.2023.1124295)
Supplement: Supplementary file 1 [file Data_Sheet_1.PDF]

## Additional File

Intervention mapping, Step 3: Theory-based methods and applications to achieve the change objectives

| **Determinant:** Knowledge - *Individual level – Behavior*<br>*Behavioral outcome 1*: Establish a healthy sleep pattern | | |
|---|---|---|
| **Change objectives** | **Methods** | **Application** |
| P.O 1 Select pattern of sleep relevant to lifestyle<br>K.1 Develop knowledge about the most common pattern of sleep (1.1-1.2) | 1.1 Consciousness raising<br>1.2Using imagery | 1.1 Use of guideline about the common pattern of sleep<br>1.2 Use of image with the relation between the amount of sleeping time and mood (tired, very tired, rested) |
| P.O 2 Select physical activities relevant to lifestyle<br>K.2 Develop knowledge about the benefits of physical activities to improve sleep (1.3-1.5) | 1.3 Consciousness raising<br>1.4 Using imagery<br>1.5 Belief selection | 1.3 Use of guideline about the benefits of physical activities (paper or media)<br>1.4 Use of image with the effects of physical activities on the body (paper or media)<br>1.5 Use of videos/newsletters about the relation between physical activities and improvement of sleep |
| P.O 3 Monitor progress in increasing sleep<br>K.3 Develop knowledge about own pattern of sleep (1.6) | 1.6 Self-monitoring of behavior | 1.6 Use of a journal by the client to register sleeping pattern |
| P.O 4 Cope with the challenges of engaging in selected pattern of sleep<br>K.4 Develop knowledge of the challenges to achieve a good pattern of sleep and safer places to sleep (1.7-1.10) | 1.7 Consciousness raising<br>1.8 Active learning<br>1.9 Mobilizing social support<br>1.10 Reinforcement | 1.7 Discussion about the challenges to achieve a good pattern of sleep<br>1.8 Development of an individual list (paper) pointing the challenges<br>1.7;1.9 Provision of a list with safer places to sleep (locations and contacts)<br>1.10 Discussion about how safer places contribute to a healthy sleeping pattern |
| *Behavioral outcome 2:* Establish a healthy Diet | | |
| **Change objectives** | **Methods** | **Applications** |
| P.O 1 Select pattern of eating relevant to lifestyle<br>K.1 Develop knowledge about the possible pattern of eating in their lifestyle (1.1-1.2) | 1.1 Consciousness raising<br>1.2Using imagery | 1.1 Use of a guideline about common eating patterns<br>1.2 Use of images with the food that the individual can eat each day |
| P. O 2 Monitor food intake<br>K.2 Develop knowledge about a healthier pattern of eating (1.3 - 1.4) | 1.3 Self- monitoring of behavior<br>1.4 Feedback | 1.3 Use of a journal by the client to register the food intake<br>1.4 Discussion about the expected healthy eating habit and the real individual habit |

| | | |
|---|---|---|
| P.O 3 Cope with the challenges of engaging in selected pattern of eating<br>K.3 Develop knowledge about the places to eat healthily (1.5 - 1.7) | 1.5 Consciousness raising<br>1.6 Feedback<br>1.7 Active learning | 1.5 Development of a list with some triggers that cause bad consumption of food<br>1.7 Discussion about the challenges to achieve a healthy pattern of eating<br>1.7 Development of an individual list pointing the individual`s challenges<br>1.5 Use of a list with the places to eat<br>1.6 Feedback about healthy places to eat |
| *Behavioral outcome 3:* Manage cocaine use | | |
| **Change objectives** | **Methods** | **Applications** |
| P.O1 Monitor Cocaine use<br>K.1 Develop knowledge about the habitual consumption of cocaine and its characteristics (1.1 -1.3) | 1.1 Consciousness raising<br>1.2 Self-monitoring of behavior<br><br>1.3 Using Imagery | 1.1; 1.2 Use of a journal to register cocaine and other psychoactive substances commonly used together<br>1.3 Use of image with a measurement of cocaine (one rock, 250 grams)<br>1.3 Use of image with measurement of other psychoactive substance (alcohol)<br>1.1; 1.3 Development of knowledge about the pathways of cocaine by image and video<br>1.1; 1.3 Development of knowledge about the pathways of cocaine and other psychoactive substance by image and video |
| P.O 2 Cope with the challenges of manage cocaine use<br>K. 2 Develop knowledge about the challenges to manage consumption of cocaine (1.4-1.6) | 1.4 Consciousness raising<br><br><br>1.5 Active learning<br><br><br><br><br><br>1.6 Self-monitoring of behavior | 1.4 Development of a list with some triggers to consumption of cocaine<br>1.4;1.5 Discussion about the challenges to achieve less consumption of cocaine<br>1.4; 1.5 Development of an individual list pointing the individual`s challenges<br>1.4 ;1.6 Use of a journal to register cocaine use with description of place, time, and usual company for consumption |
| *Behavioral outcome 4:* Manage anxiety | | |
| **Change objectives** | **Methods** | **Applications** |
| P.O 1 Become *aware* of and name anxiety<br>K.1.Develop knowledge about what anxiety means (1.1 – 1,3) | 1.1 Consciousness raising about anxiety<br><br><br><br>1.2 Awareness of anxiety - Peplau`s theory – Nursing Verbal Interventions | 1.1 Use of images or videos presenting neurophysiological effects of anxiety related with body reactions<br><br>1.2 Use of questions to the individual: "Are you anxious?"; "Are you nervous?"; "Are you nervous |

| | | |
|---|---|---|
| | 1.3 Providing cues | now?"; "Are you upset?"; "Are you tense now?"<br><br>1.3 Use of a comparable fictional case of anxiety if the client is in denial.<br>Use of questions in 1.4 below if client recognizes their anxiety. |
| P.O2 Become aware of and state the connection between the anxiety and behaviors used to relieve it<br>K.2 Develop knowledge about the connection between the anxiety and behaviors used to relieve it (1.4 – 1.7) | 1.4 Syntax - Peplau`s theory – Nursing Verbal Interventions<br><br>1.5 Consciousness raising-<br>1.6 Self-reevaluation<br><br>1.7 Environmental reevaluation | 1.4 Use of questions to realize the connection between anxiety and behavior used to relieve it: "What are you doing now to relieve being nervous?".<br>If the client says: "nothing", the nurse may ask: "What do you usually do to get comfortable?"; "When upset in the past, what did you do then?"<br>1.5,1.6 Development of list with the characteristics and names of behaviors<br>1.7 Encouragement of client to describe how their family members (or peers, friends) feel about these behaviors |
| P.O 3 Identify the expectations created<br>K.3 Develop knowledge about the influence of expectations (1.8 – 1.9) | 1.8 Identification - Peplau`s theory – Nursing Verbal Interventions<br><br>1.9 Elaboration – Peplau's theory – Nursing Verbal Interventions | 1.8 Use of questions to identify the expectations that increase anxiety: "What were you thinking about before you felt upset?"<br>1.9 Use of questions to elaborate the meaning of expectations: "What expectations?"; "Origins?"; "How long held?"; "how important?"; "Can they be changed or given up?"; "Was the expectation reasonable capable of fulfillment?".<br>Addition of questions to identify the discomfort felt: "In what part of the body did you experience discomfort?"; "How much discomfort?" |
| P.O 4 Recognize the connection between unfulfilled expectations and what happened instead<br>K.4 Develop knowledge about the connection between unfulfilled expectations and what happened instead (1.10 – 1.12) | 1.10 Consciousness raising<br><br>1.11 Syntax<br><br>1.12 Identification | 1.10 Development of list with the expectations<br>Use of questions in 1.11 below if the client has formulated the expectations.<br>1.11 Use of questions to stimulate the connection between the expectations and what happened instead: "What happened instead? When? How? Where?"; "Is there someone related to the expectation?"; "What |

| | | |
|---|---|---|
| | | interfered in the achievement of expectations?”;<br>Use of questions to identify the relief behaviors when the expectation is not achieved: “What relief behaviors are used when the expectations are not achieved?<br><br>1.12 Use of questions to identify the connection between relief behaviors and relief patterns when the expectations are not achieved:<br>- “Are there connection between relief behaviors and the relief patterns?” |
| P.O 5 Consider which factors in the sequence are amenable to control<br>K.5 Develop knowledge about the connection between the controllable factors and anxiety (1.13 – 1.16) | 1.13 Consciousness raising<br>1.14 Reckoning<br>1.15 Active learning<br>1.16 Environmental reevaluation | 1.13 Development of list with factors that can be amenable<br>1.14 Revision of the expectation in relation to what is possible<br>1.15 Discussion about what change might be possible, after the client identify and control some factors in the situation that happened instead of expectations, through their questions: “ What do you think you will feel when the expectations don`t occur?<br>1.16 Development of list with the discomfort felt, and relief behaviors |

| **Determinant:** Knowledge – *Interpersonal level – Environmental*<br>*Interpersonal outcome:* Decrease social isolation and loneliness | | |
|---|---|---|
| **Change objectives** | **Methods** | **Applications** |
| P.O 1 Identify different places to reduce loneliness and social isolation<br>K. 1 Develop knowledge about accessible locations for social interaction near the place where the client lives (1.1 – 1.3) | 1.1 Consciousness raising<br><br>1.2 Pros and cons<br><br>1.3 Tailoring | 1.1 Use of list with different healthy places<br>1.2 Development of list to compare advantages and disadvantages about each place presented<br>1.3 Provision of maps with the location of places and routes |
| P.O 2 Contact family<br>K.2 Develop knowledge about family history (1.4 -1.5) | 1.4 Empathic linkage<br>1.5 Feedback | 1.4 Careful listening of client’s family history<br>1.5 Feedback about the main points of client’s family history |
| P.O 3 Establish network with other people<br>K. 3 Develop knowledge about the importance of healthy network (1.6) | 1.6 Elaboration | 1.6 Development of a link among the connection of unhealthy network, increase in anxiety, and |

| | | |
|---|---|---|
| | | maintenance of bad relief behaviors |
| P.O 4 Establish contact with people who can help them with job opportunities<br>K.4 Develop knowledge about the importance of a network that can help them with job opportunities (1.7 -1.8) | 1.7 Elaboration<br>1.8 Social skills training | 1.7 Development of knowledge about the importance of healthy friendships to achieve better job opportunities<br>1.8 Teaching of effective social interaction in specific situations (e.g: job interviews) through techniques of behavior rehearsal.<br>1.8Development of a fictional situation for the client imagine their reaction to the situation. |
| P.O 5 Be more physically active<br>K. 5 Develop knowledge about physical activity programs in different places near the place they live (1.9 – 1.10) | 1.9 Elaboration<br>1.10 Pros and cons | 1.9 Use of a list with different places to practice physical activities<br>1.10 Discussion about the pros and cons of each place presented |
| *Interpersonal outcome 2:* Establish good relationships with friends, peers and family | | |
| **Change objectives** | **Methods** | **Applications** |
| P.O 1 Evaluate past, current, and potential relationships<br>K.1 Develop knowledge about the past, present and future relationships (1.1- 1.4) | 1.1 Elaboration<br>1.2 Providing cues<br>1.3 Environmental reevaluation<br>1.4 Restructuring the social environment | 1.1 Discussion about the relationships developed<br>1.2 Development of a comparison between characteristics of relations in the past and the present<br>1.3 Encouragement to describe:<br>- How their family members (or peers, friends) feel about their older relational bonds<br>- How their family members (or peers, friends) feel about their present relational bonds<br>1.3 Discussion about the reaction of their friends, peers and family to the past and present relationships<br>1.4 Encouragement to identify barriers to start new relationships. |
| P.O2 Avoid complicated relationships with friends, peers and family<br>K.2 Develop knowledge and strategies about how to avoid complicated relationships<br>(1.5) | 1.5 Elaboration | 1.5 Construction of strategies about possible actions to avoid complicated relationships |
| **Determinant:** knowledge – *Organizational level – Environmental*<br>*Organizational outcome:* Leave homelessness | | |
| **Change objectives** | **Methods** | **Applications** |
| P.O 1 Find a shelter<br>K.1 Develop knowledge about the shelter available in their environmental scenario and the shelter's rules (1.1 – 1.3) | 1.1 Elaboration<br>1.2 Pros and cons<br>1.3 Using imagery | 1.1Use of a list with different shelters<br>1.2 Discussion about pros and cons of shelters |

| | | |
|---|---|---|
| | | 1.3 Provision of maps with the location of places and routes |
| P.O 2 Keep contact with their family<br>K.2 Develop knowledge about the importance of family (1.4) | 1.4 Elaboration | 1.4 Development of a connection between the lack of family`s contact with homelessness situation |
| **Determinant:** knowledge – _Community level – Environmental_<br>_Community Outcome:_ Establish psychosocial rehabilitation | | |
| **Change objectives** | **Methods** | **Applications** |
| P.O 1 Avoid dangerous places or places that remind them of harmful behavioral patterns<br>K.1 Develop knowledge about the advantages of avoiding dangerous places or places that remind them of harmful behavioral patterns (1.1- 1.4) | 1.1 Elaboration<br>1.2 Providing cues<br>1.3 Tailoring<br>1.4 Reinforcement | 1.1 Development of a connection among dangerous place, increase of anxiety and marginalization<br>1.2 Development of a connection between dangerous places and bad relief behaviors<br>1.3; 1.4 Use of documentaries about the relation of dangerous place and increase of marginalization |
| P.O 2 Develop knowledge about social support to homeless people<br>K.2 Develop knowledge about the social support available in their city (1.5 – 1.7) | 1.5Written presentation<br>1.6 Verbal presentation | 1.5 Use of a list with contact of social support to homeless people<br>1.6 Encouragement to contact social support |
| P.O 3 Develop knowledge about policies for the homeless and people who have substance use disorders<br>K. 3 Develop knowledge about the specific policies for the homeless and people who have substance use disorders available in their city (1.8 – 1.9) | 1.8 Using imagery<br>1.9 Mobilizing social support | 1.8; 1.9 Provision of information about specific policies to homeless and people who have substance use disorders |

| **Determinant:** Triggers - _Individual level – Behavior_<br>_Behavioral outcome 1_: Establish a healthy sleep pattern | | |
|---|---|---|
| **Change objectives** | **Methods** | **Applications** |
| P.O 1 Select pattern of sleep relevant to lifestyle<br>T.1 Analyze the triggers that interfere with sleep (2.1 - 2.3) | 2.1 Consciousness raising<br>2.2 Active learning<br>2.3 Elaboration | 2.1;2.2 Use of a list containing triggers that interfere with sleep<br>2.3 Discussion to identify triggers that interfere in the sleep and provide alternatives for inhibit these triggers (menu of options). |
| P.O 2 Select physical activities relevant to lifestyle<br>T. 2 Identify triggers that interfere with physical activity (2.4 – 2.5) | 2.4 Consciousness raising<br>2.5 Restructuring the physical environment | 2.4 Development of a link between triggers (frequency and probability) with absence of physical activity<br>2.5 Encouragement to identify barriers for starting a new exercise regime (e.g., lack of motivation, and discuss ways in which they could help |

| | | |
|---|---|---|
| | | overcome them – e.g., going to the park to do exercise with a friend) |
| P.O 3 Monitor progress in increasing sleep<br>T.3 Evaluate triggers that interrupt sleep | 2.6 Consciousness raising | 2.6 Provision of a list with triggers that interrupt sleep |
| P.O 4 Cope with the challenges of engaging in selected pattern of sleep<br>T.4 Avoid triggers that interrupt sleep (2.7 – 2.9) | 2.7 Planning coping response<br>2.8 Mobilizing social support<br>2.9 Feedback | 2.7; 2.8 Provision of social support to face their trigger<br>2.9 Provision of a positive reinforcement to change sleeping pattern |
| *Behavioral outcome 2:* Establish a healthy diet | | |
| **Change objectives** | **Methods** | **Applications** |
| P.O 1 Select pattern of eating relevant to lifestyle<br>T.1 Analyze triggers that interfere in the healthy diet (2.1 – 2.2) | 2.1 Consciousness raising<br><br>2.2 Active learning | 2.1 Use of a list with possible triggers that interfere in the healthy diet<br>2.2 Adaptation of the list for their reality |
| P.O 2 Monitor food intake<br>T.2 Evaluate the triggers that affect the diet | 2.3 Active learning | 2.3 Discussion about the triggers that affect the client's diet |
| P.O 3 Cope with the challenges of engaging in selected pattern of eating<br>T.3 Avoid the triggers that interfere with healthy eating | 2.4 Reinforcement | 2.4 Provision of positive reinforcement to face the triggers that interfere in the healthy eating |
| *Behavior outcome 3*: Manage cocaine use | | |
| **Change objectives** | **Methods** | **Applications** |
| P.O 1 Monitor cocaine use<br>T.1 Analyze the triggers that interfere directly or indirectly in cocaine use (2.1 – 2.3) | 2.1 Consciousness raising<br>2.2 Personalized risk<br><br>2.3 Relapse Prevention | 2.1 Identification of specific triggers that generate the need to use cocaine<br>2.2 Analysis of all the factors involved with the triggers<br>2.3 Development of strategies to avoid environmental triggers |
| P.O 2 Cope with the challenges of manage cocaine use<br>T.2 Avoid the triggers that influence in the cocaine use (2.4 – 2.7) | 2.4 Planning coping response<br>2.5 Mobilizing social support<br>2.6 Avoidance/ reducing exposure to triggers for the cocaine use<br>2.7 Restructuring the social environment | 2.4; 2.5 Provision of social support to face the trigger present in the cocaine use<br>2.6 Avoidance of the situations that provide triggers to cocaine use<br>2.7 Change (if possible) of their usual social environment |
| *Behavior Outcome 4:* Manage anxiety | | |
| **Change objectives** | **Methods** | **Applications** |
| P.O 1 Become aware of and name anxiety<br>T.1 Identify the triggers of anxiety (2.1 – 2.2) | 2.1Consciousness raising<br>2.2 Active learning | 2.1 Development of a list with triggers that are related with anxiety<br>2.2 Establishment of a connection between the triggers in the list with the triggers in their life that generate anxiety |

| | | |
|---|---|---|
| P.O2 Become aware of and state the connection between the anxiety and behaviors to relieve it<br>T.2 Define the connection between triggers and anxiety (2.3 – 2.4) | 2.3 Consciousness raising<br>2.4 Environmental reevaluation | 2.3 Development of a list with characteristics of the triggers (when, where, who)<br>2.4 Encouragement to describe if client's family members (or peers, friends) identify triggers that precede anxiety |
| P.O 3 Identify the expectations created<br>T.3 Analyze the triggers involved in the operative expectations (2.5 – 2.7) | 2.5 Consciousness raising<br>2.6;2.7 Active learning | 2.5; 2.6 Definition of the triggers after the client identified the expectation and relief behaviors by brainstorming<br>2.6 Provision of a list with the triggers related with operative expectations<br>2.7 Development of a table relating the operative expectations and the triggers |
| P.O 4 Recognize the connection between unfulfilled expectations and what happened instead<br>T.4 Appraise the connection among triggers, expectations and what happened instead (2.8) | 2.8 Elaboration | 2.8 Development of an observation and consensual validation of variants of relief behaviors |
| P.O 5 Consider which factors in the sequence are amenable to control<br>T.5 Categorize the factors in possible triggers for anxiety (2.9 – 2.10) | 2.9 Personalized risk<br><br><br><br><br>2.10 Planning coping responses | 2.9 Instruction to avoid situations that can generate their problematic triggers<br>2.10 Formulation of strategies to avoid these situations |

| **Determinant:** Relief Behaviors - <u>*Individual level – Behavior*</u><br><u>*Behavioral outcome 1:*</u> Establish a healthy sleep pattern | | |
|---|---|---|
| **Change objectives** | **Methods** | **Applications** |
| P.O 1 Select pattern of sleep relevant to lifestyle<br>RB.1 Create new relief behavior to achieve sleep (3.1 – 3.3) | 3.1 Consciousness raising<br><br>3.2Active learning<br><br><br>3.3Tailoring | 3.1 Emphasis on the importance of Mindful Breathing to achieve sleep<br>3.2 Development of knowledge about how to take a deep breath before sleeping (Step 3 in the mindful breathing)<br>3.3 Discussion with the client about their vulnerable living situation, due to the lack of safety when sleeping on the street<br>3.4 Teaching of the adaptation of mindful breathing to be awakened by signals of danger. |
| P.O 2 Select physical activities relevant to lifestyle<br>RB.2 Identify physical activity as a relief behavior (3.4 – 3.5) | 3.4 Consciousness raising<br>3.5 Using imagery | 3.4; 3.5 Provision of information through pamphlets, video, pictures, with the benefits of physical activity to |

| | | |
|---|---|---|
| | | decrease anxiety (body relaxation) |
| P.O 3 Monitor progress in increasing sleep<br>RB.3 Distinguish the relief behaviors that promote healthy sleeping habits from those that impede them (3.6) | 3.6 Active learning | 3.6 Development of a table relating the relief behaviors that promote healthy sleeping habits from those that impede them |
| P.O 4 Cope with the challenges of engaging in selected pattern of sleep<br>RB.4 Modify the relief behaviors that are used to achieve sleep (3.7) | 3.7 Active learning | 3.7 Use of a table developed in 3.6 to replace the harmful relief behaviors. |
| *Behavioral outcome 2:* Establish a healthy diet | | |
| **Change objectives** | **Methods** | **Applications** |
| P.O 1 Select pattern of eating relevant to lifestyle<br>RB.1 Develop new relief behavior to achieve a good diet (3.1 – 3.2) | 3.1 Counterconditioning<br>3.2 Cue altering | 3.1; 3.2 Replacement of unhealthy behavior for healthy behavior (e.g., scheduling a regular time to eat, avoiding alcohol before a meal) |
| P.O 2 Monitor food intake<br>RB.2 Distinguish the relief behaviors that promote from those that impede a healthy diet (3.3) | 3.3 Active learning | 3.3 Development of a table distinguishing the relief behaviors that promote from those that impede a healthy diet |
| P.O 3 Cope with the challenges of engaging in selected healthy eating pattern<br>RB.3 Modify the relief behaviors to achieve a healthy diet (3.4) | 3.4 Active learning | 3.4 Use of the table developed in 3.3 to replace the harmful relief behaviors |
| *Behavior outcome 3:* Manage cocaine use | | |
| **Change objectives** | **Methods** | **Applications** |
| P.O 1 Monitor cocaine use<br>RB.1 Develop new relief behaviors (3.1 - 3.2) | 3.1 Counterconditioning<br>3.2 Consciousness raising | 3.1; 3.2 Discussion about other relief behaviors that offer pleasure similar to cocaine (mindfulness, physical activity, food etc.) |
| P.O 2 Cope with the challenges of manage cocaine use<br>RB.2 Modify the relief behaviors (3.3 – 3.4) | 3.3 Counterconditioning<br><br>3.4 Environmental reevaluation | 3.3 Engagement with the new relief behaviors instead of cocaine<br>3.4 Verification of the effects of the engagement with the new relief behaviors to reduce cocaine consumption (replacement and/or reduction of drug use for damage control) |
| *Behavior Outcome 4*: Manage anxiety | | |
| **Change objectives** | **Methods** | **Applications** |
| P.O 1 Become aware of and name anxiety<br>RB.1 Identify the relief behaviors used during anxiety (3.1 – 3.2) | 3.1 Consciousness raising<br><br>3.2 Elaboration | 3.1 Identification of the relief behaviors<br>3.2 Connection of anxiety and relief behaviors |

| | | |
|---|---|---|
| P.O 2 Become aware of and state the connection between the anxiety and behaviors to relieve it<br>RB.2 Analyze the relief behaviors (3.3 – 3.4) | 3.3 e 3.4 Elaboration | 3.3 Identification of the part of the body where the discomfort is felt through an illustration<br>3.4 Development of an analysis of their relief behaviors through the use of questions:<br>- Is there a series of relief behaviors that are used?<br>- Does the series recur in the same order in subsequent anxiety- producing behaviors? |
| P.O 3 Identify the expectations created<br>------------------------------------------------------------- | ------------------------------------------------------------------------------------------------------------------------------------- | -------------------------------------------------------------------------------------------------------------- |
| P.O 4 Recognize the connection between unfulfilled expectations and what happened instead<br>RB. 4 Identify whether the relief behaviors are connected with the expectations (3.5) | 3.5 Possession | 3.5 Recognition of the connection between the relief behaviors and the expectations to reduce physical and emotional anxiety |
| P.O 5 Consider which factors in the sequence are amenable to control<br>RB.5 Modify the relief behaviors that are changeable (3.6) | 3.6 e 3.7 Self-reevaluation | 3.6 Development of a list with the relief behaviors used and highlight those which can be changed.<br>3.7 Joint construction of a set of alternative relief behaviors |

| **Determinant:** Self-efficacy/ Self- esteem and Skills - <u>*Individual level – Behavior*</u><br><u>*Behavioral outcome 1*</u>: Establish a healthy sleep pattern | | |
|---|---|---|
| **Change objectives** | **Methods** | **Applications** |
| P.O 1 Select pattern of sleep relevant to lifestyle<br>SES. 1 Build self-confidence in ability to adequate pattern of sleep in their lifestyle (4.1 – 4.2) | 4.1 Guided practice<br><br>4.2 Self-monitoring of behavior | 4.1 Provision of a feedback about client's breathing performance as they apply the techniques to improve their sleep (mindfulness, deep breathing)<br><br>4.2 Use of a journal by the client to register how the techniques influence their pattern of sleep over time |
| P.O 2 Select physical activities relevant to lifestyle<br>SES. 2 Build self-confidence in ability to do physical activity (4.3 – 4.6) | 4.3 Goal setting<br><br>4.4 Self-monitoring of behavior<br>4.5 Planning coping response | 4.3 Discussion of the goal for the next meeting, deciding on a target that is feasible in their lifestyle to improve their physical activity.<br>4.4 Use of a journal by the client to register physical activities done over time.<br>4.5 Development of a list of potential constraints and brainstorming on how to overcome those, e.g. If the park |

| | | |
|---|---|---|
| | | that the client uses to exercise is closed, they can find other places.<br>4.5 Discussion on the importance of physical activities to reduce anxiety and improve sleep. |
| P.O 3 Monitor progress in increasing sleep<br>SES. 3 Build self-confidence in ability to change pattern of sleep (4.7 – 4.8) | 4.7 Improving physical and emotional states<br><br>4.8 Cue altering | 4.7 Emphasis on the importance of deep breathing to relax before sleep.<br>4.8 Discussion about their vulnerable living situation, due to the lack of safety when sleeping on the street<br>4.9 Teaching of what an unconscious message is:<br>- Before sleeping breathe deeply while keeping in mind the sign that can alert them of danger during sleep[1]<br><br>4.8 Encouragement to search for safer places to sleep avoiding disturbances |
| P.O 4 Cope with the challenges of engaging in selected pattern of sleep<br>SES. 4 Build self-confidence in ability to face the challenges of adopting healthy sleeping pattern | 4.9 Cue altering<br>4.10 Mobilizing social support<br><br>4.11 Planning coping response | 4.9 Encouragement to search for a place that might improve the pattern of sleep<br>4.10 Use of a list with social support that provides free places to sleep, such as homeless shelter<br>4.11 Development of a list with the potential challenges to healthy patterns of sleep and ways to overcome them |
| *<u>Behavioral outcome 2:</u>* Establish a healthy diet | | |
| **Change objectives** | **Methods** | **Applications** |
| P.O 1 Select pattern of eating relevant to lifestyle<br>SES.1 Build self-confidence to adjust eating patterns to their lifestyle (4.1) | 4.1 Reinforcement | 4.1 Use of positive messages to demonstrate that the client can achieve healthy eating patterns |
| P.O 2 Monitor food intake<br>SES. 2 Build self- confidence to monitor one`s own food intake (4.2) | 4.2 Self-monitoring of behavior | 4.2 Use of a diary by the client to register their food intake |
| P.O 3 Cope with the challenges of engaging in selected healthy eating patterns | 4.3 Cue altering<br>4.4 Mobilizing social support | 4.3 Encouragement to search for a place that provides healthy food |

[1] An example of unconscious message is when a mother wakes up instantly when she hears her baby cry

| | | |
|---|---|---|
| SES. 3 Build self-confidence to face the challenges of adopting healthy eating patterns (4.3- 4.5) | 4.5 Planning coping response | 4.4 Use of a list with social support that provides free or low-cost healthy food.<br>4.5 Development of a list with potential barriers and means to overcome them |
| *Behavior outcome 3* : Manage cocaine use | | |
| **Change objectives** | **Methods** | **Applications** |
| P.O 1 Monitor cocaine use<br>SES.1 Build self-confidence to monitor one`s own cocaine use (4.1 – 4.2) | 4.1 Self-reevaluation<br>4.2 Self-monitoring of behavior | 4.1; 4.2 Use of a diary by the client to register their cocaine intake and the habitual form of consumption |
| P.O 2 Cope with the challenges of manage cocaine use<br>SES.2 Build self-confidence to generate coping strategies to deal with the challenges (4.3 – 4.5) | 4.3 Cue altering<br>4.4 Mobilizing social support<br><br>4.5 Planning coping response<br><br>4.6 Relapse Prevention | 4.3 Encouragement to search for a place with limited access to cocaine<br>4.4 Development of a list with social support that provides other relief behaviors instead of cocaine use<br>4.6 Encouragement to go to the gym to overcome anxiety, instead of use cocaine<br>4.6 Development of a list with potential barriers and means to overcome them.<br>4.7 Development of strategies to avoid environmental triggers |
| *Behavior Outcome 4*: Manage anxiety | | |
| **Change objectives** | **Methods** | **Applications** |
| P.O 1 Become aware of and name anxiety<br>SES. 1 Build self-confidence to recognize anxiety (4.1) | 4.1 Verbal persuasion | 4.1 Use of positive messages to show that the client is capable of recognizing anxiety |
| P.O2 Become aware of and state the connection between the anxiety and behaviors to relieve it<br>SES.2 Build self-efficacy to establish connection between anxiety and relief behaviors (4.2-4.3) | 4.2 Self-monitoring behavior<br>4.3 Alignment | 4.2; 4.3 Use of an illustration to identify the part of body where the discomfort is experienced or use of question to identify the part of body where the discomfort is experienced:<br>- What part of the body? |
| P.O 3 Identify the expectations created<br>SES. 3 Build self-confidence to recognize expectations (4.4) | 4.4 Verbal persuasion | 4.4 Use of positive messages to show that the client is capable of recognizing expectations |
| P.O 4 Recognize the connection between unfulfilled expectations and what happened instead<br>SES. 4 Build self-efficacy to identify the connection between expectations, what happened instead, anxiety and relief behaviors (4.5) | 4.5 Syntax | 4.5 Building of connections among expectations held, the reality, anxiety and relieve behaviors |
| P.O 5 Consider which factors in the sequence are amenable to control | 4.6 Guided practice | 4.6 Use of all models to target behaviors that reduce anxiety (deep breath; mindfulness; physical activity; healthy diet) |

| | | |
|---|---|---|
| SES. 5 Build self-confidence to change expectations and factors to break the cycle (4.6) | | |
| **Determinant:** Self-efficacy/ Self- esteem and Skills – *<u>Interpersonal level – Environmental</u>*<br>*<u>Interpersonal outcome 1</u>*: Decrease social isolation and loneliness | | |
| **Change Objectives** | **Methods** | **Applications** |
| P.O 1 Identify different places to reduce loneliness and social isolations<br>SES. 1 Build self-efficacy to recognize barriers` to knowing different places (2.1) | 2.1 Changing routine | 2.1 Advisement of means to change routine daily or weekly to limit exposure to behavioral cues, and to create an opportunity to meet other people |
| P.O 2 Contact family<br>SES.2 Build self-confidence to establish contact with their family (2.2) | 2.2 Empathic linkage | 2.2 Encouragement to contact their family through empathic linkage developed between nurse and client |
| P.O 3 Establish network with other people<br>SES. 3 Build self-confidence to establish healthy networks | ----------------------------------------<br>----------------------------------------<br>----------------------------------------<br>------------------ | --------------------------------------<br>--------------------------------------<br>--------------------------------------<br>-- |
| P.O 4 Establish contact with people who can help them with job opportunities<br>SES. 4 Build self-confidence to contact people who can help them with job opportunities | ----------------------------------------<br>----------------------------------------<br>----------------------------------------<br>----------------------------------------<br>----------------------------------------<br>---------------------------------------- | --------------------------------------<br>--------------------------------------<br>--------------------------------------<br>--------------------------------------<br>--------------------------------------<br>------------- |
| P.O 5 Be more physically active<br>SES. 5 Build self- confidence to practice physical activity (2.4) | 2.3Action planning | 2.3 Encouragement to plan physical activities at a regular rate<br>2.3 Encouragement to engage in physical activities to overcome anxiety |
| *<u>Interpersonal outcome 2</u>* : Establish good relationships with friends, peers and family | | |
| **Change objectives** | **Methods** | **Applications** |
| P.O 1 Evaluate past, current, and potential relationships<br>SES. 1 Build self-confidence to differentiate good and complicated relationships | ----------------------------------------<br>----------------------------------------<br>-- | --------------------------------------<br>--------------------------------------<br>-------- |
| P.O 2 Avoid complicated relationships with friends, peers and family<br>SES. 2 2 Build self-confidence in their ability to change/ finish problematic relationships (2.1 – 2.3) | 2.1 Counterconditioning<br>2.2 Pros and cons<br>2.3 Stimulus control | 2.1; 2.2 Development of a list with pros and cons of the problematic relationship<br>2.3 Development of new habits to avoid problematic people (e.g. change routes, change of places they attend) |
| **Determinant:** Self-efficacy/ Self- esteem and Skills – *<u>Organizational level – Environmental</u>*<br>*<u>Organizational outcome</u>*: Leave homelessness | | |
| **Change Objectives** | **Methods** | **Applications** |
| P.O 1 Find a shelter<br>SES. 1 Build self-confidence to find a better shelter for their reality (2.1 – 2.2) | 2.1 Feedback<br>2.2 Pros and cons | 2.1 Positive reinforcement to find a shelter<br>2.2 Development of a list with pros and cons of each shelter |
| P.O 2 Keep contact with their family<br>SES. 2 Build self-confidence to keep contact with their family and return to their | 2.3 Pros and cons | 2.3 Development of a list with pros and cons of returning to their family`s house |

| | | |
|---|---|---|
| family`s house, if possible<br>(2.3) | | |
| **Determinant:** Self-efficacy/ Self- esteem and Skills – *<u>Community level – Environmental</u>*<br>*<u>Community Outcome</u>*: Establish psychosocial rehabilitation | | |
| **Change Objectives** | **Methods** | **Applications** |
| P.O 1 Avoid dangerous places or places that remind them of harmful pattern behavior<br>SES. 1 Express self-efficacy to deal with environmental cues (2.1 – 2.2) | 2.1 Restructuring the social environment<br>2.2; 2.3 Stimulus control | 2.1 Identification of barriers to reinsertion in society<br>2.2 Discussion of socially undesirable habits<br>2.3 Development of new habits |
| P.O 2 Develop knowledge about social support to homeless people<br>SES. 2 ----------------------------------------------- | --------------------------------------------------------------------------------------------------------------------------------------------- | ---------------------------------------------------------------------------------------------------------------- |
| P.O 3 Develop knowledge about policies for the homeless and people who have substance use disorders<br>SES. 3 --------------------------------------------------- | --------------------------------------------------------------------------------------------------------------------------------------------- | ---------------------------------------------------------------------------------------------------------------- |

| | | |
|---|---|---|
| **Determinant:** Relations– *<u>Interpersonal level – Environmental</u>*<br>*<u>Interpersonal outcome1 :</u>* Decrease social isolation and loneliness | | |
| **Change objectives** | **Methods** | **Applications** |
| P.O 1 Identify different places to reduce loneliness and social isolations<br>R.1 Establish interpersonal relations in new places (3.1) | 3.1 Social skills training | 3.1 Use of a simulation to practice effective social interaction through techniques of behavior rehearsal. |
| P.O 2 Contact family<br>R.2 Prepare to reestablish relational bonds (3.2 – 3.3) | 3.2 Environmental reevaluation<br>3.3 Social skills training | 3.2 Use of questions to establish feelings of family members (or peers, friends) about their previous relational bonds:<br>- How do your family members (or peers, friends) feel about your previous and current relational bonds?<br>3.3 Use of simulation to practice reestablishing relational bonds through techniques of behavior rehearsal |
| P.O 3 Establish network with other people<br>R.3 ------------------------------------------------------------ | ------------------------------------------------------------------------------------------------------------------------------------------------------- | ---------------------------------------------------------------------------------------------------------------- |
| P.O 4 Establish contact with people who can help them with job opportunities<br>R.4 -------------------------------------------------------------- | ------------------------------------------------------------------------------------------------------------------------------------------------------------------------ | ------------------------------------------------------------------------------------------------------------------------ |

| | | |
|---|---|---|
| | ------------------------------------------------------- | ------------------------------------------------ |
| P.O 5 Be more physically active<br>R.5 Establish relational ties to support physical activity (3.4) | 3.4 Restructuring the social environmental | 3.4 Identification of barriers to start a new exercise e.g., lack of motivation<br>3.4 Discussion of means to help them overcome these barriers e.g., going to the gym with a friend |
| *Interpersonal outcome 2* : Establish good relationships with friends, peers and family | | |
| **Change Objectives** | **Methods** | **Applications** |
| P.O 1 Evaluate past, current, and potential relationships<br>R. 1 Deep understanding about interpersonal relations that are significant (relational bonds and ties) (3.1 – 3.3) | 3.1 Consciousness raising<br>3.2 Pros and cons<br>3.3 Environmental reevaluation | 3.1 Conversation about the client' relations.<br>3.2 Identification of pros and cons in the relationships<br>3.3 Use of questions to establish feelings of family members (or peers, friends) about their current relational bonds:<br>- How do your family members (or peers, friends) feel about your current relational bonds? |
| P.O 2 Avoid complicated relationships with friends, peers and family<br>R.2 Recognize complicated relational bonds and ties (3.4 – 3.5) | 3.4 Pros and cons<br>3.6 Stimulus control | 3.4 Identification of pros and cons of complicated relational bonds<br>3.5 Discussion of the connection between complicated bonds, increase in anxiety, and bad relief behaviors |
| **Determinant:** Relations – *Organizational level – Environmental*<br>*Organizational outcome* : Leave homelessness | | |
| **Change Objectives** | **Methods** | **Applications** |
| P.O 1 Find a shelter<br>R.1 Identify shelter as a positive relational tie | 3.1 Elaboration | 3.1 Encouragement to stay in shelter and establish relations with other people that share the same reality |
| P.O 2 Keep contact with their family<br>R.2 ------------------------------------------------------------------- | ----------------------------------------------------------------------------------------------------------------------------------------------------------------------------------------- | ------------------------------------------------------------------------------------------------------------------------------------------------------ |
| **Determinant:** Relations – *Community level – Environmental*<br>*Community Outcome*: Establish psychosocial rehabilitation | | |
| **Change Objectives** | **Methods** | **Applications** |
| P.O Avoid dangerous places or places that remind them of harmful pattern behavior<br>R.1 --------------------------------------------------------------- | ----------------------------------------------------------------------------------------------------------------------------------------------------------------------------------------- | ------------------------------------------------------------------------------------------------------------------------------------------------------ |
| P.O 2 Develop knowledge about social support to homeless people<br>R.2 --------------------------------------------------------------- | ---------------------------------------------------------------------------------------------------------------- | ---------------------------------------------------------------------------------------- |

| | | |
|---|---|---|
| P.O 3 Develop knowledge about policies for the homeless and people who have substance use disorders<br>R.3 ---------------------------------------------------------------------- | ------------------------------------------------------------------------------------------------------------------------------------------------------------------------------------------------------------ | ------------------------------------------------------------------------------------------------------------------------------------------------- |
