# Supplementary material for: An anxiety management intervention for people with substance use disorders (ITASUD): An intervention mapping approach based on Peplau's theory
Source: Front Public Health. 2023 Feb 21;11:1124295. doi: 10.3389/fpubh.2023.1124295 (PMC9989484; doi:10.3389/fpubh.2023.1124295)
Supplement: Supplementary file 2 [file Data_Sheet_2.PDF]

Additional file 2

FOCUS GROUPS WITH NURSES - INTERVIEW GUIDE

Objectives

The objectives of the focus group are as follows:

- To assess attitudes towards components of the intervention to achieve behavioural (health sleep patterns, health eating, management of cocaine intake) and environmental outcomes (decrease of social isolation, reinsertion in the society) to determine their feasibility and acceptability; and
- To identify understanding/perceptions, barriers and enablers to providing the intervention.
- To identify potential clinician-related barriers to the implementation fidelity of this intervention

Programme

Part A: Overview and Introductions (10 minutes)
Part B: Intervention structure and content (30 minutes)
Part C: Summary and next steps (5 minutes)

<u>Part A: Introduction</u> (approx. 10 mins)

1. **Refreshments**

2. **Overview** (approx. 2 minutes)
    - Brief explanation of the purpose of the overall study and focus group
    - Overview of the structure of the focus group
    - Explanation of ground rules
        - Recording for transcription
        - One person speaking at a time
        - Confidentiality
        - Consent

3. **Introductions** (approx. 4 minutes)
    - Researcher`s name
    - Name and past or current experience of individual interpersonal relation between nurse and client for the management of anxiety with cocaine user.
        - Do you have any past or current experience of providing interpersonal relation for clients (drug users) with anxiety?

4. **Discussion** (approx. 4 mins)
    - What is your general experience and views of interpersonal relation approaches for anxiety in cocaine users?
        - Successes, challenges
        - How did you motivate clients to attend, stay in the group?

Part B: Intervention Structure and Content (approx. 30 mins)

1. **Presentation of Structure & Content of the intervention** (approx. 10 mins)
    - Aim of the intervention – To equip cocaine users with strategies to manage anxiety
    - Brief overview of evidence-base underpinning intervention
    - Population – cocaine users with anxiety
    - Structure
        - Individual-based – delivered to individual
        - Nurse led
        - Once time
    - 8 minutes of nurse-led group discussion and problem solving session with supporting hand outs regarding components to achieve behavioural and environmental outcomes for achieve the management of anxiety
        - Topics include: behavioural factors (physical recommendations, healthy eating, healthy pattern of sleep, and managing cocaine intake); and environmental factors (strategies to decrease social isolation, to reinsert clients into society, to get out clients from homeless situation).
    - Any specific questions for clarification?

2. **Discussion Topics (approx. 20 mins)**

HIGH PRIORITY

- What are your initial impressions about the structure and content of the intervention? (2 mins)

- What do you think about the feasibility of this intervention within your service delivery context? (3 mins)

- Is there any (other) specific feedback you have on the feasibility of implementing this intervention in your service setting with these clients? (10 mins)
    - Population (Demand) – sufficient number of appropriate clients, cocaine/crack users - How many demand is likely to exist?
    - Structure – 1 week, 5 times during the week, balance of appointment (Acceptability)- (20- 30 mins) - The process is attractive to program deliverers? If not , what is the suggestions to improve the program? How we can do more attractive?
    - Knowledge / Skills to facilitate intervention (Adaptation) - The manual of intervention is clear enough to understanding? The applications reach the patients? Is it an easy format? If not, what are the suggestions to improve the intervention?

    - Facilities and resources (Practicality)- (appropriate facilities, equipment, staffing) – The intervention fit with organizational culture (time of appointment, location of appointment, nurse sufficient to do the intervention)? If not, what are the suggestions to improve the fit?

  - Of all the challenges you have mentioned, which ones are the biggest or most important? How might the most important challenges be addressed?

LOWER PRIORITY

- What resources or support do you feel you would require to successfully provide this intervention? (5 mins)
    - Is there anything you would need to feel confident providing equip to manage anxiety in cocaine in an interpersonal relationship format and covering the topics specified (physical recommendations, healthy eating, healthy pattern of sleep, managing cocaine intake; and strategies to decrease social isolation, to reinsert clients into society, to get out clients from homeless situation)
  - Which, if any of these resources/supports, are essential – for example you could not provide this intervention without them?

<u>Part C: Summary and next steps</u> (5 minutes)

1.Discussion

So our aim is to go away from here and adapt the intervention based on your feedback to ensure that it is acceptable to you and feasible to be implemented within the settings where you are working.

Coding Framework for Feasibility Analysis

**Intervention Components**		**Experiences & Attitudes**	
		**Negative**	**Positive**
Programme Participants	• Cocaine/crack users	Cocaine/crack users -	Cocaine/crack users +
Programme Content/determinants	• knowledge<br>• triggers<br>• relief behaviors<br>• self-efficacy<br>• relations	knowledge -<br>triggers -<br>relief behaviors-<br>self-efficacy-<br>relations-	knowledge +<br>triggers +<br>relief behaviors +<br>self-efficacy+<br>relations+
Programme Structure	• Individual<br>• 1 Week<br>• 5 days consecutively<br>• 1 = 30 min<br>• 2 -5 = 20 min	Individual-<br>1 Week-<br>5 days consecutively -<br>1= 30min-<br>2-5 =20min-	Individual+<br>1 Week +<br>5 days consecutively +<br>1= 30min+<br>2-5 =20min +
Programme Delivery	• Staffing (no. of nurses, involvement, admin)<br>• Facilities/Equipment	Staff-<br><br>Facilities-	Staff+<br><br>Facilities+

<u>Close of focus group</u>

If anyone want to say something, ideas, complain, or any questions about the study. You're welcome to contact me individually at any time. Thank you again for your time and we look forward to working with you.
